# CT-based multimodal deep learning for non-invasive overall survival prediction in advanced hepatocellular carcinoma patients treated with immunotherapy

**DOI:** 10.1186/s13244-024-01784-8

**Published:** 2024-08-26

**Authors:** Yujia Xia, Jie Zhou, Xiaolei Xun, Jin Zhang, Ting Wei, Ruitian Gao, Bobby Reddy, Chao Liu, Geoffrey Kim, Zhangsheng Yu

**Affiliations:** 1 Department of Bioinformatics and Biostatistics, School of Life Sciences and Biotechnology, Shanghai Jiao Tong University, Shanghai, China; 2 SJTU-Yale Joint Center for Biostatistics and Data Science, School of Life Sciences and Biotechnology, Shanghai Jiao Tong University, Shanghai, China; 3 Department of Statistics, School of Mathematical Sciences, Shanghai Jiao Tong University, Shanghai, China; 4 Statistics in Global Statistics and Data Science, Beigene, Shanghai, China; 5 PiHealth USA, Cambridge, MA USA; 6 Clinical Research Institute, Shanghai Jiao Tong University School of Medicine, Shanghai, China

**Keywords:** Unresectable hepatocellular carcinoma, Computed tomography, Prognostic predictions, Multi-modal, Deep learning

## Abstract

**Objectives:**

To develop a deep learning model combining CT scans and clinical information to predict overall survival in advanced hepatocellular carcinoma (HCC).

**Methods:**

This retrospective study included immunotherapy-treated advanced HCC patients from 52 multi-national in-house centers between 2018 and 2022. A multi-modal prognostic model using baseline and the first follow-up CT images and 7 clinical variables was proposed. A convolutional-recurrent neural network (CRNN) was developed to extract spatial-temporal information from automatically selected representative 2D CT slices to provide a radiological score, then fused with a Cox-based clinical score to provide the survival risk. The model’s effectiveness was assessed using a time-dependent area under the receiver operating curve (AUC), and risk group stratification using the log-rank test. Prognostic performances of multi-modal inputs were compared to models of missing modality, and the size-based RECIST criteria.

**Results:**

Two-hundred seven patients (mean age, 61 years ± 12 [SD], 180 men) were included. The multi-modal CRNN model reached the AUC of 0.777 and 0.704 of 1-year overall survival predictions in the validation and test sets. The model achieved significant risk stratification in validation (hazard ratio [HR] = 3.330, *p* = 0.008), and test sets (HR = 2.024, *p* = 0.047) based on the median risk score of the training set. Models with missing modalities (the single-modal imaging-based model and the model incorporating only baseline scans) can still achieve favorable risk stratification performance (all *p* < 0.05, except for one, *p* = 0.053). Moreover, results proved the superiority of the deep learning-based model to the RECIST criteria.

**Conclusion:**

Deep learning analysis of CT scans and clinical data can offer significant prognostic insights for patients with advanced HCC.

**Critical relevance statement:**

The established model can help monitor patients’ disease statuses and identify those with poor prognosis at the time of first follow-up, helping clinicians make informed treatment decisions, as well as early and timely interventions.

**Key Points:**

- An AI-based prognostic model was developed for advanced HCC using multi-national patients.
- The model extracts spatial-temporal information from CT scans and integrates it with clinical variables to prognosticate.
- The model demonstrated superior prognostic ability compared to the conventional size-based RECIST method.

**Graphical Abstract:**

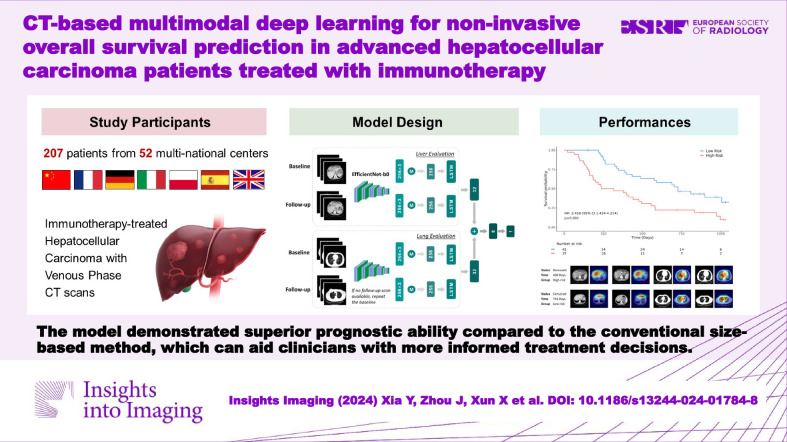

## Introduction

Hepatocellular carcinoma (HCC), the predominant histologic type of liver cancer, represents aggressive malignancy and high lethality [1], and is the third leading cause of cancer-related death [2]. Patients with advanced HCC are often assessed as unsuitable for surgery. Such patients usually underwent palliative operations or were treated with nonsurgical systemic approaches, including chemotherapy, radiation, targeted therapy, and immunotherapy [3, 4]. Unfortunately, these treatments offer a poor prognosis, with a median overall survival (OS) of approximately 1-year [5, 6]. Monitoring a patient’s disease status and identifying those with poor prognoses can aid clinicians in making informed treatment decisions and timely interventions.

Response evaluation criteria in solid tumors (RECIST) serves as a standardized method for evaluating oncologic treatment responses in clinical practices [7, 8]. It provides guidelines for measuring changes in tumor size on longitudinal radiographic imaging, and categorizes the treatment outcomes into four classes: complete response, partial response, stable disease, or progressive disease [9]. While disease progression is commonly associated with a poorer prognosis, it has not been definitively established that there is a strictly positive correlation between the two [10]. Therefore, the traditional guideline does not provide a reliable prediction of the OS of HCC patients in clinical practice.

Currently, the rising popularity of artificial intelligence has led to the widespread use of convolutional neural networks (CNN) to extract features from clinical images automatically and provide insight into disease prognosis [11, 12]. Many deep learning models exist to predict survival outcomes in HCC patients using pathological images, primarily Hematoxylin and Eosin slides [13–15]. Additionally, gene sequencing has been utilized to differentiate survival subpopulations of HCC patients [16]. These quantitative imaging studies all achieved favorable performances in forecasting the prognostic risk. However, single time-point biomarkers may be less effective in advanced stages, due to the lack of pathological and genetic data in some patients. Serial CT scans offer a non-invasive and informative approach to capturing tumor dynamics. However, to the best of our knowledge, there is currently no prognostic prediction model available that utilizes serial CT scans for advanced HCC.

Therefore, we aimed to develop a deep-learning model to predict survival outcomes for advanced HCC patients. The study data comprises a multi-center clinical trial cohort with patients previously treated with conventional therapies before participating in the immunotherapy trial. We collected the longitudinal CT imaging and a few clinical variables to construct the multi-modal prognostic model. The designed model utilized a convolutional-recurrent neural network (CRNN) structure to decode spatial information from CT images and extract temporal information of tumor changes between the baseline and the follow-up. Various prediction models with different input modalities were compared. To evaluate the added benefit provided by the proposed model, risk stratification ability was compared between the deep learning approach and the traditional RECIST criteria.

## Materials and methods

### Data collection

**Fig. 1:**
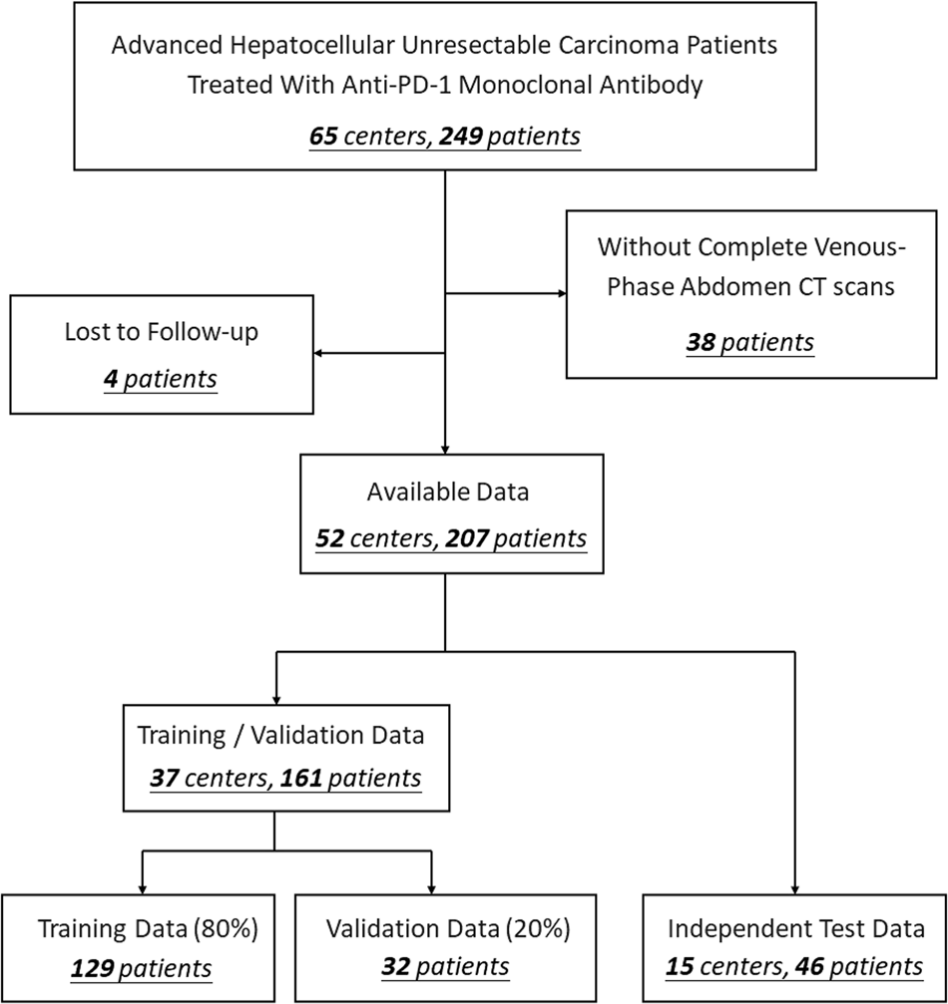
Workflow of the patient selection process

All data passed ethics review (application no. I2021173I) and were approved by the Human Genetic Resource Administration of China (approval no. [2021] GH5565), with all participants providing informed consent. This retrospective, multi-centered study includes patients with unresectable HCC treated with anti-PD-1 monoclonal antibodies (clinicaltrials.gov number: NCT03419897) from April 9, 2018 to July 6, 2022. Patients’ CT scans and clinical information were collected. Exclusion criteria were: (1) lack of complete abdominal CT scans on the venous phase and (2) loss to follow-up. After the sample selection, 207 patients from 52 multi-national centers (sites located in China, France, Germany, Italy, Poland, Spain, and the United Kingdom) were used to train and validate the model. Patients were randomly assigned by center for model development (comprising 161 patients from 37 centers) and evaluation (comprising 46 patients from 15 centers). The dataset for model development was further randomly divided into training and validation sets in an 8:2 ratio (Fig. 1).

### CT imaging and clinical information acquisition

Patients were required to take oral or intravenous contrast before enhanced CT scans. Enhanced abdominal imaging was mandatory for all participants, and chest imaging was strongly recommended. The imaging methodology was kept consistent across visits for a patient (i.e., acquisition time for each phase, contrast agent, scan mode, and parameters). Venous-phase CT images were acquired using various scanners and then resampled and linearly interpolated to 5-mm section thickness. The abdomen scans were normalized to window width (WW) 400, window level (WL) 0, and chest scans were normalized to WW 1200, WL −600.

**Fig. 2:**
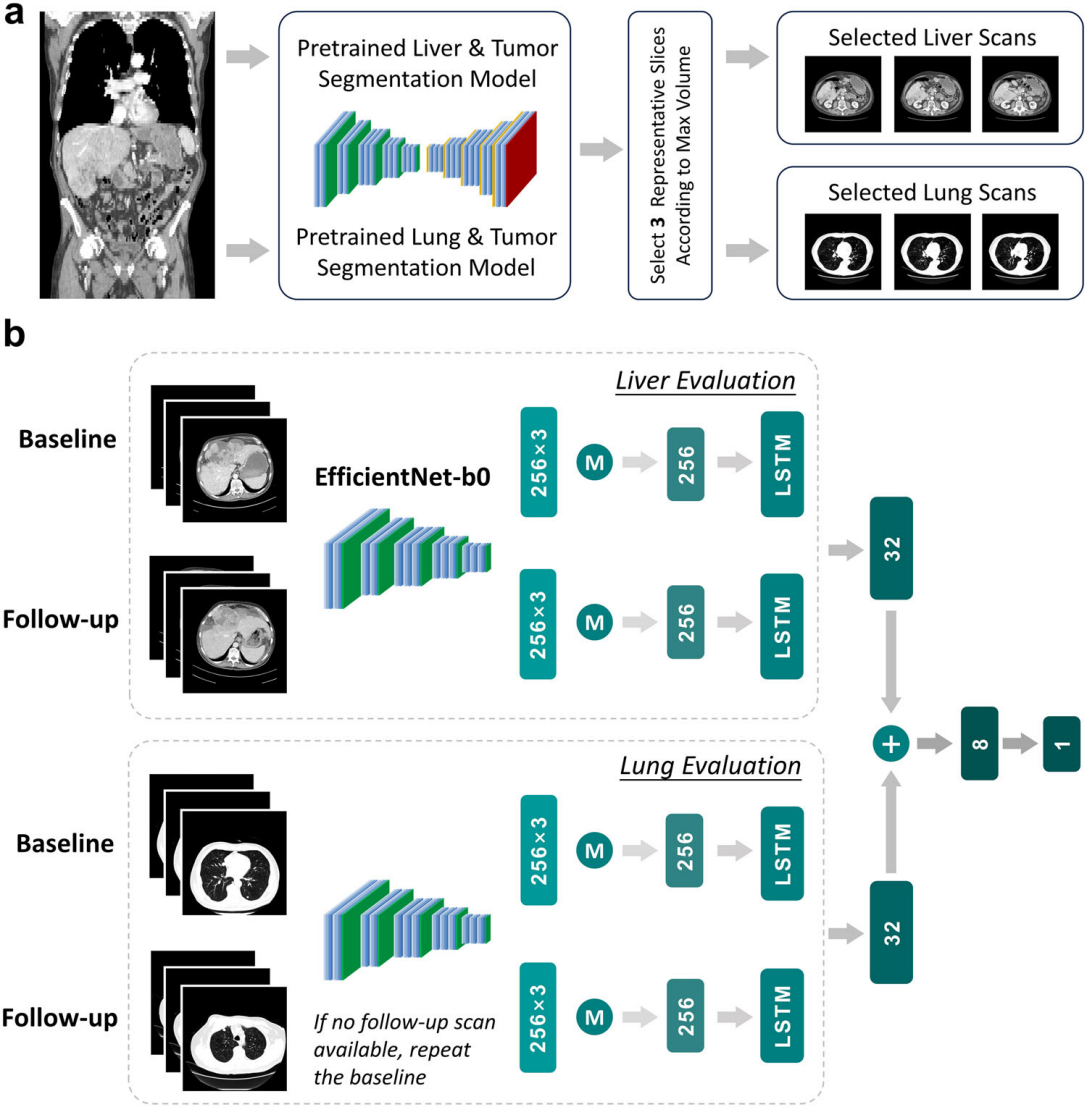
Image selection and model illustration. **a** The process of representative CT image selection, from 3D scans to selected 2D liver and lung scans. **b** The flowchart for the deep learning model. Feature dimensions are marked. M, taking the average

To reduce computational load while keeping the most informative parts of whole CT scans, three liver slices and three lung slices from each 3D CT scan were automatically selected as the input for the model. The selection process for representative slices of the liver and lung is as follows: If a tumor is present, three 2D slices with the top three largest tumor sizes in the axial view were chosen from the 3D scans. If there are fewer than three slices containing tumors, slices with tumors were first selected, then the remaining slices were selected based on the largest organ size (either liver or lung) to present the overall state of that specific organ. If there is no tumor present, all representative slices were selected based on the largest organ size (Fig. 2a). To achieve this, four pre-trained automated models were utilized to segment the boundaries of the lung, lung tumors, liver, and liver tumors. These pretrained segmentation models are based on nnU-Net architecture [17], which is a self-adaptive framework that can automatically adjust model hyperparameters based on dataset characteristics to achieve optimal performance. It has achieved state-of-the-art (SOTA) results in multiple organ and tumor segmentation tasks including liver and lung [18]. These selected 2D CT images were reshaped to 224 × 224 and standardized to the mean of zero and variance of one to serve as the input for the deep learning model. The training images were randomly flipped with a probability of 0.5 for data augmentation.

The clinical data comprised of seven variables: tumor histological differentiation type, presence of non-alcoholic steatohepatitis (NASH) or non-alcoholic fatty liver disease (NAFLD), prior surgical history, presence of partial or complete portal vein tumor thrombosis (PVTT), treatment of external beam radiation therapy (EBRT), transarterial embolization (TAE)/transarterial chemoembolization (TACE), and radiofrequency ablation (RFA)/microwave ablation (MWA).

The study label, i.e., the OS, was retrieved from the clinical recorded form (CRF). For censored patients, censor time was defined as the maximum value between the time recorded in the CRF and the time of the latest CT scan.

### Model development

Figure 2b presents the workflow of the deep learning model (‘Rad-D’ in the subsequent content) to process radiological images. The model inputs consist of the baseline and first follow-up scan. Every patient has the baseline and the follow-up abdominal scan as part of the inclusion criteria and imaging acquisition protocols. Whereas, if follow-up chest images are unavailable, the baseline image would be replicated and used as a substitute for the follow-up, which is a common practice referred to as the last observation carried forward (LOCF) strategy [19] in longitudinal data analysis. LOCF is a method for imputing missing data in a dataset, which involves replacing any missing values with the last known, non-missing value for that data point. LOCF can increase the utilization of data without increasing the complexity of the model.

A total of 12 images, with three images for each organ at each time point, were fed into EfficientNet-b0 [20] with weights pretrained on ImageNet to extract features. EfficientNet-b0 is a lightweight CNN backbone that adopted compound scaling to enhance performance. The extracted features of the three representative images from both baseline and follow-up were averaged and then fed into the long short-term memory (LSTM) module [21] to capture the temporal information. LSTM is a type of recurrent neural network (RNN) that leverages memory cells and gating mechanisms to effectively capture and process sequential data. Then, the liver and lung features were concatenated, and linear layers were utilized to predict the risk score.

Brief descriptions of models with different input modalities are listed here:

CLN: a multivariate Cox proportional hazard model performs risk regression using seven clinical variables (Fig. S1a).

Rad-S: a deep learning model without follow-up scans and the RNN layers. It only used EfficientNet-b0 to extract features from baseline images (Fig. S1b).

RadCLN-S: a multi-modal model that combines the risk score from CLN and Rad-S and then uses a bivariate Cox model to calculate to predict the prognostic risk (Fig. S1c).

Rad-D: the above-mentioned deep learning model that assessed both baseline and follow-up CT scans using CRNN structure.

RadCLN-D: a multi-modal model that combines the clinical risk score from CLN and the radiological score from the output of Rad-D by using a bivariate Cox model to predict the prognostic risk (Fig. S1d).

The deep learning model’s gradient optimization process adopted the loss function of partial likelihood, which is an unbiased and efficient way to estimate the parameters of the Cox proportional hazards model.

The model was trained 200 epochs using 1 × NVIDIA A100 (40 G), with the CRNN implemented in PyTorch 1.11.0 and MONAI 0.9.1, utilizing a batch size of 16, and the Adam optimizer with a learning rate of 5 × 10^−5^. The code is available at https://github.com/EstelleXIA/ProgHCC.

### Evaluation metrics and statistical analyses

Model performance was evaluated using Harrell’s concordance index (*C*-index) [22] and the time-dependent area under the receiver operating characteristic curves (AUCs) at different time points [23]. *C*-index quantifies the model’s capability to correctly rank the relative risks of pairs of individuals. Survival estimates were calculated using the Kaplan–Meier method for low and high-risk groups, which were stratified based on the median prediction score of the training set. Hazard ratios (HR) were further computed and the significance was measured using the log-rank test. For the RadCLN-D model, Cox regression coefficients were used to generate a nomogram. Calibration curves were utilized to show concordance between actual and predicted outcomes determined by the nomogram.

Statistical tests were performed with survival, survcomp, survminer, timeROC, and rms packages in R4.2.2. A two-sided *p* < 0.05 indicated statistical significance. Model interpretation used Gradient-weighted Class Activation Mapping (Grad-CAM) [24] and was visualized by pytorch-gradcam 0.2.1.

## Results

### Patient characteristics

Characteristics of the 207 patients (mean age, 61 years ± 12 [SD], 180 male) can be found in Table 1. Among all patients, the median interval between baseline and follow-up CTs is 55 days. The median survival time is 475 days, in which 138 patients (66.7%) have deceased. There was no significant difference in survival status among the training, validation, and test datasets (Fig. S2, train vs validation, HR, 1.051, 95% confidence interval [CI]: 0.662–1.669, *p* = 0.833; train vs test, HR, 1.032, 95% CI: 0.686–1.552, *p* = 0.880; validation vs test, HR, 1.039, 95% CI: 0.604–1.790, *p* = 0.889). For histological types, out of 207 patients, 29 (14%) were highly differentiated, 154 (74%) were moderately differentiated, 23 (11%) were low differentiated, and 1 (< 1%) was undifferentiated. For the baseline symptoms, 35 (17%) had NASH/NAFLD and 36 (17%) had PVTT. For the additional treatments, 109 (53%) had undergone surgery, 10 (5%) received EBRT, 121 (58%) received TAE/TACE, and 58 (28%) received RFA/WMA. Multivariable Cox regression calculated a risk score based on the seven variables using the formula: ${\rm{Score}} =0.3747 \times {\rm{Differentiation}} + 0.1593\times {\rm{NASH|NAFLD}} \! - \!0.1801\times {\rm{Surgery}}+0.6732 \times {\rm{PVTT}}-0.8235\,\times \,{\rm{EBRT}} + 0.6482 \,\times {\rm{TAE| TACE}}-0.4497 \times {\rm{RFA|MWA}}$ (Fig. S3, *C*-index = 0.630, *p* = 0.018).

**Table 1:** Patient characteristics

Variables	Train, (*n* = 129)	Validation, (*n* = 32)	Test, (*n* = 46)
Sex			
Male	111 (86)	29 (91)	40 (87)
Female	18 (14)	3 (9)	6 (13)
Age, year	61 ± 12	61 ± 14	62 ± 10
Median interval between two scans, day	53	52	56.5
Status			
Deceased	83 (64)	23 (72)	32 (70)
Censored	46 (36)	9 (28)	14 (30)
Median survival time, day	467	475	488
Differentiation type			
Highly differentiated	18 (14)	4 (13)	7 (15)
Moderately differentiated	100 (78)	20 (63)	34 (74)
Low differentiated	10 (8)	8 (25)	5 (11)
Undifferentiated	1 (1)	0 (0)	0 (0)
NASH/NAFLD			
Yes	26 (20)	3 (9)	6 (13)
No	103 (80)	29 (91)	40 (87)
Surgery			
Yes	67 (52)	17 (53)	25 (54)
No	62 (48)	15 (47)	21 (46)
PVTT			
Yes	24 (19)	5 (16)	7 (15)
No	105 (81)	27 (84)	39 (85)
EBRT			
Yes	6 (5)	2 (6)	2 (4)
No	123 (95)	30 (94)	44 (96)
TAE/TACE			
Yes	74 (57)	20 (63)	27 (59)
No	55 (43)	12 (38)	19 (41)
RFA/WMA			
Yes	38 (29)	8 (25)	12 (26)
No	91 (71)	24 (75)	34 (74)

*NASH* non-alcoholic steatohepatitis, *NAFLD* non-alcoholic fatty liver disease, *PVTT* partial or complete portal vein tumor thrombosis, *EBRT* external beam radiation therapy, *TAE* transarterial embolization, *TACE* transarterial chemoembolization, *RFA* radiofrequency ablation, *MWA* microwave ablation

### Comparisons of different models on the survival prediction

Prediction performances were compared among CLN, Rad-S, RadCLN-S, Rad-D, and RadCLN-D on both the validation and independent test sets (Tables 2 and 3 and Fig. S4). Clinical variables displayed unfavorable prediction performances, with a *C*-index of 0.537 (95% CI: 0.406–0.668) on the validation set and 0.622 (95% CI: 0.500–0.744) on the test set. Using the baseline radiological image achieved the *C*-index of 0.692 (95% CI: 0.569–0.815) on the validation set and 0.608 (95% CI: 0.504–0.712). By incorporating the first follow-up image, performance demonstrated significant improvement, reaching 0.748 (95% CI: 0.664–0.832) and 0.681 (95% CI: 0.573–0.789) on the validation and test sets, respectively. Multi-modal inputs (RadCLN-S and RadCLN-D) outperformed the uni-modal (CLN, Rad-S, and Rad-D). RadCLN-S reached the *C*-index of 0.697 (95% CI: 0.574–0.820) on the validation set, and 0.638 (95% CI: 0.536–0.740) on the test set. RadCLN-D attained 0.752 (95% CI: 0.660–0.844) on the validation set, and 0.695 (95% CI: 0.581–0.809) on the test set. Time-dependent ROCs showed a similar pattern in survival prediction performances (Figs. S4b and S4c).

**Table 2:** Concordance index of the prognostic prediction models

	Validation		Test		Combined	
	*C*-index	*p*-value	*C*-index	*p*-value	*C*-index	*p*-value
CLN	0.537 (0.406, 0.668)	0.400	0.622 (0.500, 0.744)	0.020	0.585	0.002
Rad-S	0.692 (0.569, 0.815)	0.010	0.608 (0.504, 0.712)	0.050	0.645	0.013
RadCLN-S	0.697 (0.574, 0.820)	< 0.001	0.638 (0.536, 0.740)	< 0.001	0.662	0.075
Rad-D	0.748 (0.664, 0.832)	0.002	0.681 (0.573, 0.789)	0.020	0.729	0.310
RadCLN-D	0.752 (0.660, 0.844)	< 0.001	0.695 (0.581, 0.809)	0.004	0.734	–

Combined *C*-index is the weighted score calculated from the validation and the test set using the survcomp package in R

*p*-values of validation and test *C*-index indicate the significance of prognostic prediction (the log-rank test)

Combined *p*-value indicates the significance of the difference between the certain model and RadCLN-D (the student *t*-test)

**Table 3:** Time-dependent AUCs of the prognostic prediction models

	Validation				Test			
	0.5-year	1-year	1.5-year	2-year	0.5-year	1-year	1.5-year	2-year
CLN	0.531	0.616	0.469	0.517	0.681	0.625	0.611	0.640
Rad-S	0.839	0.768	0.765	0.740	0.741	0.673	0.594	0.581
RadCLN-S	0.831	0.782	0.761	0.755	0.788	0.709	0.617	0.632
Rad-D	0.877	0.759	0.891	0.849	0.865	0.685	0.605	0.630
RadCLN-D	0.900	0.777	0.870	0.839	0.888	0.704	0.622	0.652

**Fig. 3:**
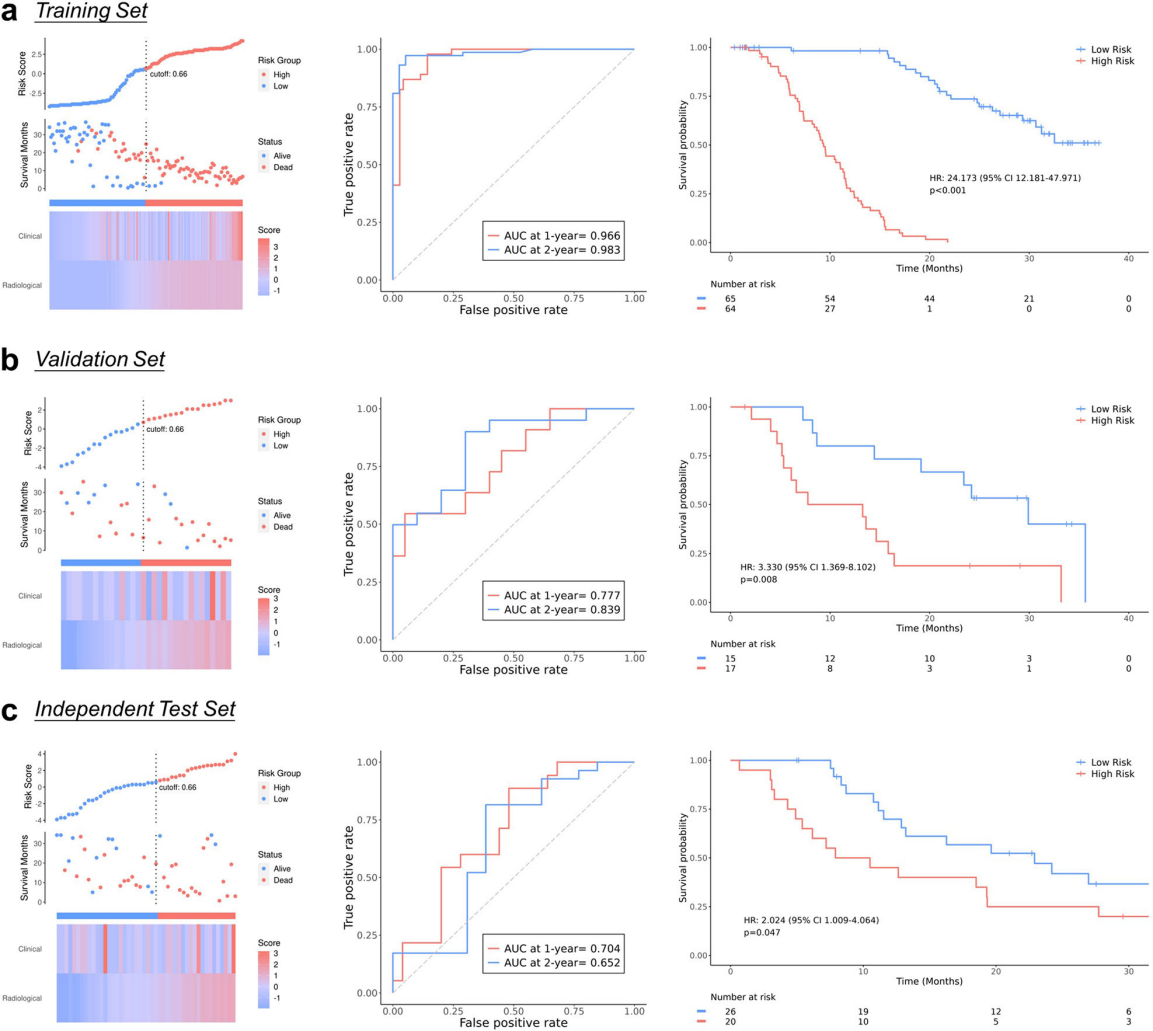
Performances of RadCLN-D. Left, the distributions of risk scores based on the multi-modal predictions are presented. Heatmaps are displayed to illustrate the distribution levels of the two modalities (radiological and clinical). Middle, time-dependent ROC curves at 1-year and 2-year. Right, Kaplan–Meier survival estimates for the OS, were stratified into low-risk and high-risk groups according to the median risk score in the training set. AUC, area under the curve; HR, hazard ratio

To further demonstrate the prognostic predictive ability of the model, the models’ capability for risk stratification was assessed. RadCLN-S, Rad-D, and RadCLN-D effectively stratified patients into high-risk and low-risk groups (Figs. 3 right, S5, and S6), demonstrating the models’ ability to identify survival risk using clinical information and baseline CT scans or solely baseline and first follow-up scans. Among all, RadCLN-D exhibited the highest predictive performance, as it included the most comprehensive information.

### RadCLN-D accurately predicts the OS

**Fig. 4:**
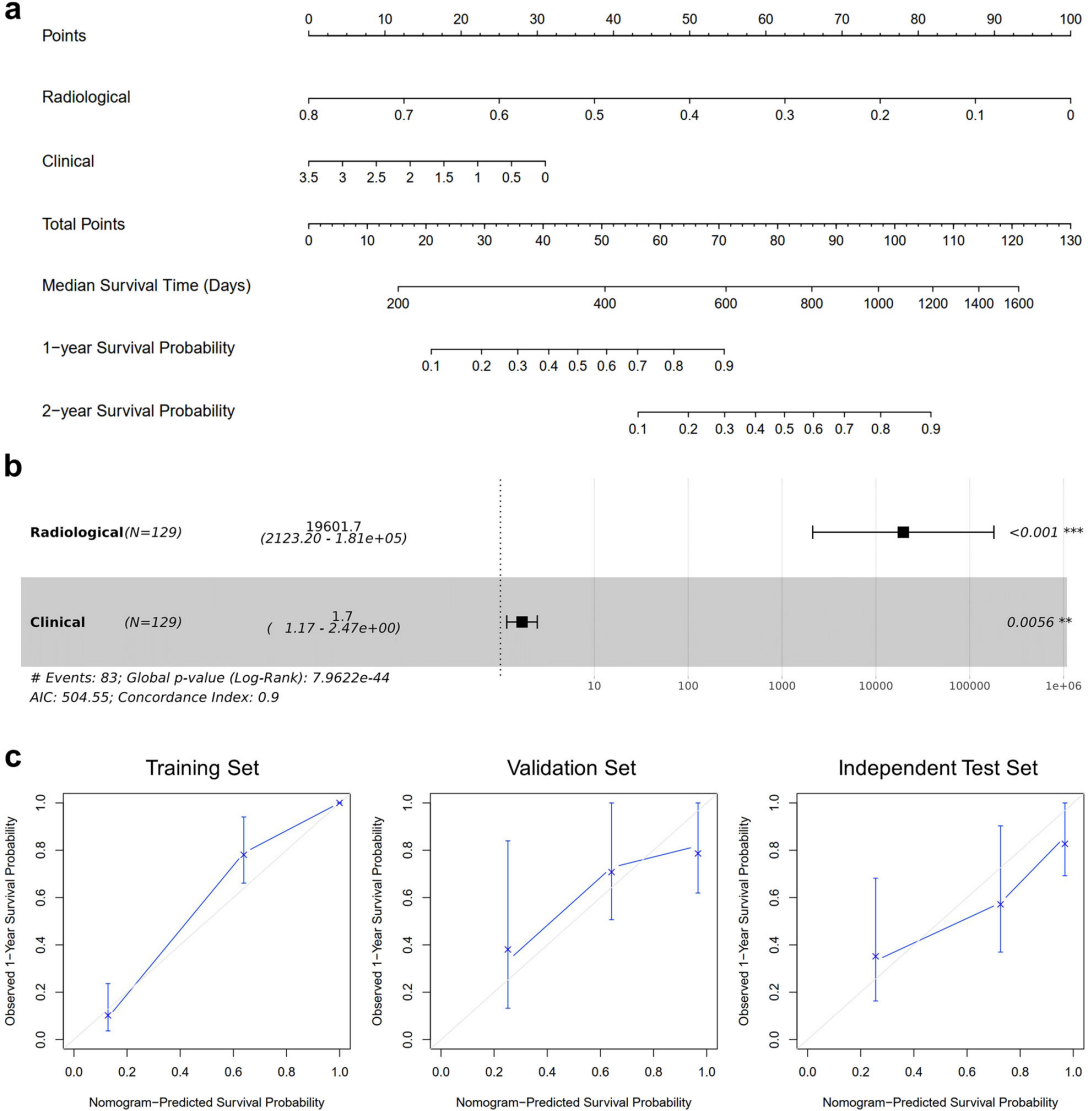
Establish and validate the nomogram of RadCLN-D. **a** For the radiological score and the clinical score, locate the corresponding value on the scale provided on the nomogram, then add up to get the total points. A vertical line from the total points value is to the predicted probability of the 1-year and 2-year survival probability. **b** Importance of the radiological score and the clinical score. **c** Calibration of the nomogram in terms of the agreement between predicted and observed 1-year survival outcomes

Performances of RadCLN-D were further detailly illustrated. RadCLN-D combines the radiological score from the output of the CRNN structure and the clinical score with the formula ${{\rm{Score}}}\,=\,9.8834\times {{\rm{Radiolog}}}{{{\rm{y}}}}_{{{\rm{score}}}}+0.5300\times {{\rm{Clinica}}}{{{\rm{l}}}}_{{{\rm{score}}}}$, the two modalities all significantly contributed to the OS prediction (Fig. 4b, radiological, *p* < 0.001; clinical, *p* = 0.0056; Wald Test). For 1-year OS predictions, the AUC is 0.966 in the training set, 0.777 in the validation set, and 0.704 in the test set. For 2-year OS predictions, the AUC is 0.983 in the training set, 0.839 in the validation set, and 0.652 in the test set (Fig. 3, middle). Patients with lower multi-modal scores tend to be censored or with a relatively longer survival time, while most patients with higher scores suffered early decease (Fig. 3, left). The median score from the training data was used to apply a cutoff for stratifying patients into high-risk and low-risk groups, i.e., ‘score > 0.66’ signifies high-risk, and ‘score ≤ 0.66’ signifies low-risk. To examine the generalizability, the risk score calculation, and cutoff stratification used in the validation and test sets were consistent with those of the training set. The multi-modal score displayed reliable predictive accuracies. It made significant risk stratifications in all the training (HR, 24.173, 95% CI: 12.181–47.971, *p* < 0.001), validation (HR, 3.330, 95% CI: 1.369–8.102, *p* = 0.008), and test sets (HR, 2.024, 95% CI: 1.009–4.064, *p* = 0.047).

A nomogram was developed based on the RadCLN-D prediction model to determine the OS for individual patients (Fig. 4a). It allows clinicians to estimate the 1-year and 2-year survival probabilities in a clear and concise manner. Calibration plots indicated favorable comparability between the nomogram and an ideal model across the training, validation, and test datasets (Fig. 4c).

Moreover, to assess the robustness of the model, the patients in the validation set and test set were grouped according to the manufacturers used. Among the 78 patients, the main manufacturers were Siemens (37 patients) and General Electric (33 patients). Due to the small sample sizes of other manufacturers such as Philips, TOSHIBA, and Hitachi Medical, these patients were not included in the analysis. The prognostic performance of patients with two major manufacturers was compared, with a *C*-index of 0.728 for the Siemens group and 0.707 for the general electric group.

### Interpretation of the deep learning model

**Fig. 5:**
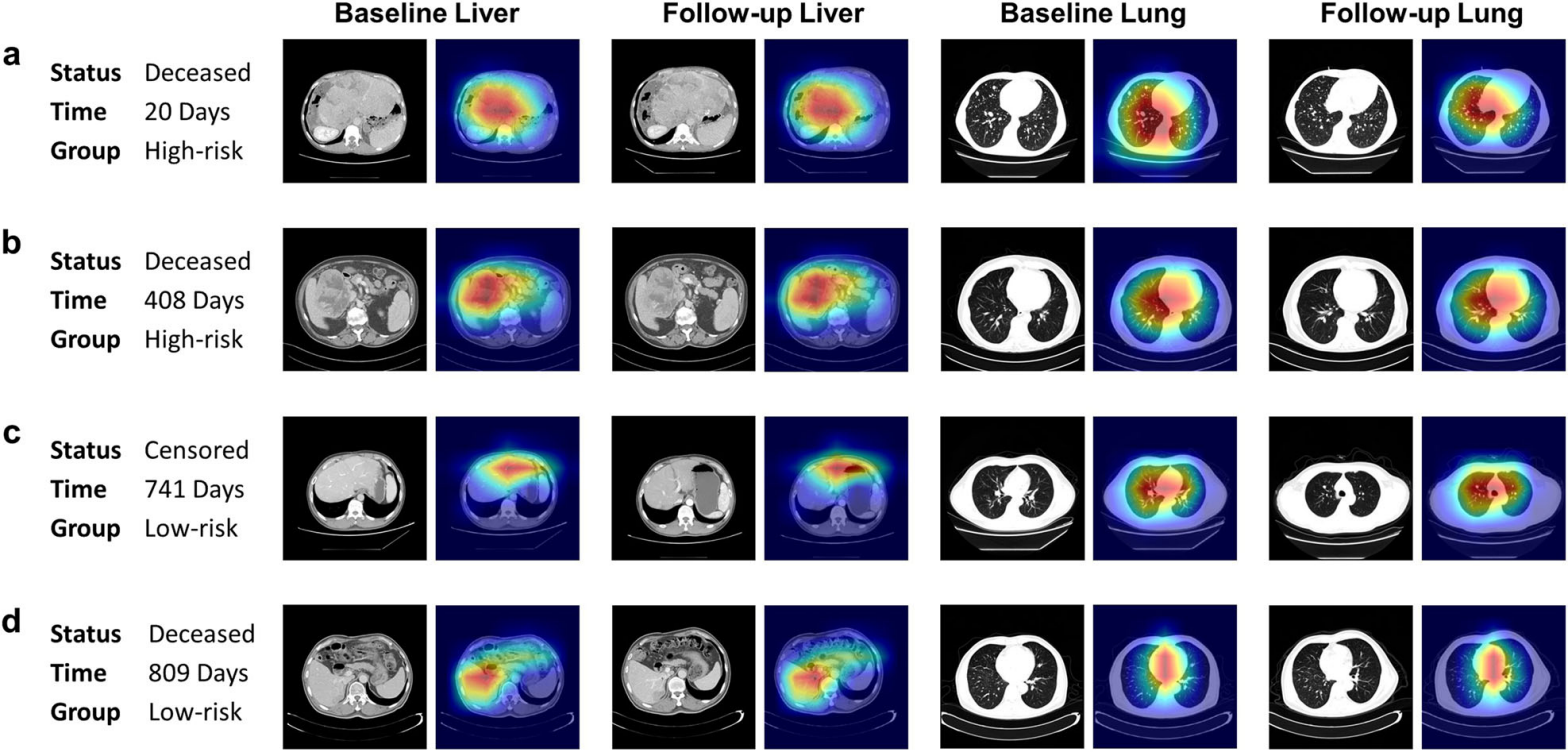
Interpretation of the deep learning model. Grad-CAM computes the gradients of the target class’s score (i.e., the risk score) with respect to the feature maps in the last convolutional layer of the network. These gradients are then weighted by the average pooling of the gradients to obtain the importance weights of the feature maps then normalized to [0,1] (with the blue color close to zero and the red color close to one) and linearly combined with the original feature maps. The four demonstrated cases are selected from the independent test set. **a**, **b** Two patients with model-predicted high-risk. **c**, **d** Two patients with model-predicted low-risk

To demonstrate the explainability of the deep learning model, four patients from the test set, including two predicted high-risk and two predicted low-risk by Rad-D, were presented to interpret the constructed CRNN architecture. Heatmaps highlighted the regions of the image that contribute most to the network’s decision-making process. The prognostic model focused particularly on the tumor regions (Fig. 5a, b, liver scans, the hottest region on the tumor), which is consistent with common medical knowledge that regions with high malignancy correlate strongly with prognosis. Non-liver-malignancies in two low-risk patients resulted in hot regions of the whole liver detected by the model (Fig. 5c, d, liver scans). Similar heatmap patterns predicted by the deep learning model can be observed in lung scans, i.e., the model focused more on suspicious lesion areas.

### Incremental value of RadCLN-D to traditional size-based method

**Fig. 6:**
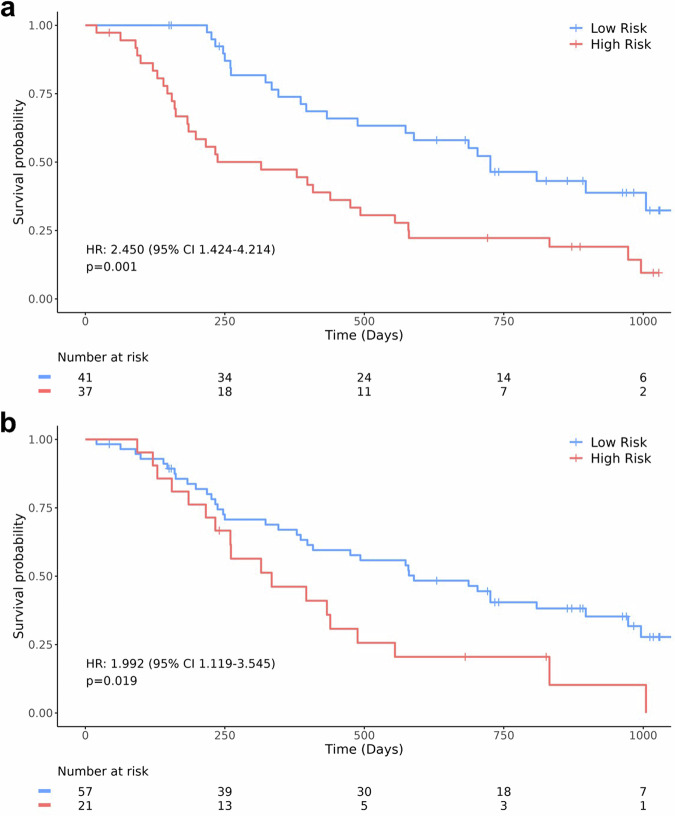
Risk stratification by the deep learning model and the conventional RECIST criteria. Results were made on the combination of the validation set and the test set. **a** Risk stratification by the deep learning model RadCLN-D, the high-risk group is defined as a score > 0.66, and the low-risk group is defined as a score ≤ 0.66. **b** Risk stratification by the RECIST criteria, the high-risk group is defined as disease progression at the first follow-up, and the low-risk is defined as no progression observed at the first follow-up. HR, hazard ratio; RECIST, response evaluation criteria in solid tumors

RECIST outcomes assessed by an independent review committee were adopted and patients with a progression status were assigned to the high-risk group. Risk stratification performances of RadCLN-D and the conventional RECIST criteria were compared (Fig. 6). RECIST outcomes showed acceptable risk prediction performance as the response status significantly stratified the high-risk group and the low-risk group (HR, 1.992, 95% CI: 1.119–3.545, *p* = 0.019). Whereas, RadCLN-D exhibited stronger categorization capability (HR, 2.450, 95% CI: 1.424–4.214, *p* = 0.001), suggesting an improvement of the deep learning-based method over the conventional size-based method.

## Discussion

In this study, we developed and validated a deep learning model that decodes spatial-temporal information from radiological imaging and clinical variables to predict the prognostic outcome of advanced HCC patients. Our multimodal approach combines the baseline and first follow-up scans with clinical information, reaching a 1-year AUC of 0.777 in the validation set and 0.704 in the independent test set. Additionally, models with missing modalities, i.e., the single-modal imaging-based model (Rad-D) and the model incorporating only baseline scans (RadCLN-S), can still achieve favorable risk stratification performance (with all *p* < 0.05, except for RadCLN-S on the test set, *p* = 0.053). Compared to conventional RECIST criteria, the deep learning model exhibits superior prognostic prediction ability (RECIST, HR, 1.992, *p* = 0.019; RadCLN-D, HR, 2.450, *p* = 0.001). This study shows that deep learning analysis of CT scans can yield valuable prognostic information to guide surveillance of immunotherapy-treated advanced HCC patients.

Prognostic prediction for HCC can help doctors formulate more targeted treatment plans and then maximize the treatment effect for patients. With the development of radiomics and deep learning techniques, there are several prognostic prediction models for HCC. He et al [25] developed a survival model for macrotrabecular-massive HCC patients using radiomics extracted from enhanced abdominal CT scans. Meng et al [26] adopted a deep learning model that utilized CNN to analyze MRI images for early recurrence prediction after hepatectomy. Xu et al [27] proposed a deep prediction network that utilizes information on both full liver and tumor masks from CT images to predict early recurrence. Zhang et al [28] used pretrained CNN models to extract features from CT scans, then employed machine learning methods to provide OS predictions in unresectable patients treated with sorafenib. Wei et al [29] proposed a deep learning model that utilized automated segmentation-based MRI radiomic signature to estimate the postsurgical early recurrence risk. However, these prognostic models have not been specifically developed for advanced HCC patients receiving immunotherapy. HCC patients treated with immunotherapy usually undergo multiple imaging follow-ups to aid in observing the efficacy of the drugs. Therefore, these models that only utilize data from a single time point, can fail to capture the dynamic growth characteristics of the tumor. Besides, the lung is the most common site of metastasis for advanced liver cancer. Therefore, evaluation of the chest condition is important for prognosis prediction. A direct application of existing methods to advanced HCC can affect the performance of the model, as they solely focus on the liver and neglect the lung.

In our developed model, we used both liver and lung images at baseline and follow-up, with a CRNN structure, to make prognostic predictions. CNNs can proficiently extract prognostically relevant features from radiological images. Integrated with clinical information, RadCLN-S enables precise survival predictions. Moreover, the RNN architecture efficiently captures tumor changes between baseline and follow-up measurements. The integration of temporal and spatial information results in improved risk stratification outcomes.

The validation results of the model performance from multiple perspectives (i.e., time-dependent AUC, risk stratification capability, and overall *C*-index) on multi-center data illustrate the practicability and superiority of the deep learning model. The comparative results of models under various input settings indicate that the model can still yield reasonably accurate predictive outcomes despite the absence of certain modalities. Furthermore, the comparison with the RECIST criteria demonstrated that, compared to the manual assessment of the growth trend of the whole-body lesions, deep learning encapsulates additional information beyond the size, adding incremental information for prognostic predictions. Our model has the potential to be effectively utilized for prognostic prediction in advanced HCC patients and aid clinicians with adaptive treatment planning to improve patient outcomes.

Our proposed prognostic model is interpretable. Heatmaps can provide a coarse location of regions relevant to prognosis, which demonstrates the interpretability of the radiological input. For heatmaps of the liver, although we did not explicitly input the liver or liver tumor region as auxiliary information in the model, the heatmaps consistently reveal highlighted areas within the liver. Specifically, in the two high-risk patients, the core areas colored the deepest red were localized to the tumor region, suggesting a strong association between the presence of the tumor and the unfavorable prognosis predicted by the model. Moreover, the model’s focus on the tumor area allows for a more comprehensive capture of tumor characteristics, such as size and morphology. These tumor-related features are closely associated with prognosis.

Besides, the chosen clinical variables in this study are also explainable. It comprises frequently used treatments for HCC, such as surgical resection and local non-surgical interventions, as well as previously established and reported prognostic variables. The results of the clinical Cox regression model (CLN model, Fig. S3) align with previous studies’ evidence. The German Cancer Research Center [30] reported that immunotherapy fails to provide a survival benefit in patients with NASH/NAFLD, owing to an accumulation of abnormal CD8 + /PD-1 + T cells within the liver. PVTT is considered a sign of advanced-stage disease, with untreated cases only having a median OS of 2.7–4.0 months [31]. Histological differentiation type is a recognized prognostic factor, with higher differentiation types typically indicating a better prognosis [32, 33]. As for treatments, patients previously treated with RFA/MWA, EBRT, or surgical resection all exhibited positive prognostic effects. There was an inverse relationship between TAE/TACE therapy and prognosis, which may be due to the higher frequency of TAE/TACE intervention in patients with more severe conditions.

Our proposed model is efficient in processing radiological imaging. The representative 2D scans were automatically selected from the original whole-body CT scans using pretrained SOTA segmentation models. Most previous works manually delineate certain tumor regions to generate the inputs [34, 35], which is labor-intensive and impracticable for advanced HCC due to the presence of multifocal hepatic lesions. Furthermore, the inclusion of the tumor and its surrounding tissue within the model inputs can provide additional indications for survival predictions. Some studies [36, 37] have employed semi-automatic seed growing to create the region of interest, but it still needs lots of human efforts in lesion identifications. Deep learning-based segmentation models can achieve considerable performance in automatically depicting tumor and tissue regions of the lung and liver [17, 38, 39]. Therefore, we incorporated the SOTA model to assist with the representative slice selection, which ultimately makes the model more user-friendly.

Our study data come from multiple centers, and the diversity of the data enables the model to be adaptive to different vendors, making it more robust and transferable across various imaging settings. Experimental results demonstrate that our model performs similarly across different devices, demonstrating the generalizability of our model.

Our study has limitations. First, some advanced liver cancer patients may have other metastatic lesions apart from the lung, such as the brain, lymph nodes, and adrenal glands. Though it has been reported that distant organ metastases, excluding pulmonary metastases, typically do not have a detrimental effect on the prognosis [40], future works can consider more metastatic organs for potential improvement of the prediction performance. Second, the study data were retrospectively collected, which can introduce various biases. Prospective studies with larger sample sizes should be conducted to further validate the practicability of the proposed model.

In conclusion, deep learning analysis of CT scans using the multi-modal CRNN model can provide valuable prognostic information, enabling the effective surveillance of patients with advanced HCC. The proposed approach could empower clinicians to make informed decisions regarding patient management and follow-up strategies based on the identified risk stratification patterns derived from the CT scans and the clinical information.

### Supplementary information

ELECTRONIC SUPPLEMENTARY MATERIAL

## Data Availability

Data generated or analyzed during the study are available from the corresponding author by request. The study source code can be found at https://github.com/EstelleXIA/ProgHCC.

## Abbreviations

AUC: Area under the receiver operating characteristic curve
CNN: Convolutional neural networks
CRNN: Convolutional-recurrent neural network
HR: Hazard ratio
HCC: Hepatocellular carcinoma
OS: Overall survival
RECIST: Response evaluation criteria in solid tumors

## References

[1] McGlynn KA, Petrick JL, El–Serag HB (2020) Epidemiology of hepatocellular carcinoma. Hepatology 73:4–13. 10.1002/hep.31288 32319693 10.1002/hep.31288 PMC7577946

[2] Siegel RL, Miller KD, Wagle NS, Jemal A (2023) CA Cancer J Clin. 10.3322/caac.21763 10.3322/caac.21763 36633525

[3] Llovet JM, Castet F, Heikenwälder M et al (2021) Immunotherapies for hepatocellular carcinoma. Nat Rev Clin Oncol 19:151–172. 10.1038/s41571-021-00573-2 34764464 10.1038/s41571-021-00573-2

[4] Bruix J, Gores GJ, Mazzaferro V (2014) Hepatocellular carcinoma: clinical frontiers and perspectives. Gut 63:844–855. 10.1136/gutjnl-2013-306627 24531850 10.1136/gutjnl-2013-306627 PMC4337888

[5] Pinter M, Scheiner B, Peck-Radosavljevic M (2021) Immunotherapy for advanced hepatocellular carcinoma: a focus on special subgroups. Gut 70:204–214. 10.1136/gutjnl-2020-321702 32747413 10.1136/gutjnl-2020-321702 PMC7788203

[6] Qin S, Ren Z, Meng Z et al (2020) Camrelizumab in patients with previously treated advanced hepatocellular carcinoma: a multicentre, open-label, parallel-group, randomised, phase 2 trial. Lancet Oncol 21:571–580. 10.1016/s1470-2045(20)30011-5 32112738 10.1016/s1470-2045(20)30011-5

[7] Jin W, Luo Q (2022) When artificial intelligence meets PD-1/PD-L1 inhibitors: population screening, response prediction and efficacy evaluation. Comput Biol Med 145:105499. 10.1016/j.compbiomed.2022.105499 35439641 10.1016/j.compbiomed.2022.105499

[8] Litière S, Collette S, De Vries EGE, Seymour L, Bogaerts J (2016) RECIST-learning from the past to build the future. Nat Rev Clin Oncol 14:187–192. 10.1038/nrclinonc.2016.195 27995946 10.1038/nrclinonc.2016.195

[9] Eisenhauer E, Therasse P, Bogaerts J et al (2009) New response evaluation criteria in solid tumours: revised RECIST guideline (version 1.1). Eur J Cancer 45:228–247. 10.1016/j.ejca.2008.10.026 19097774 10.1016/j.ejca.2008.10.026

[10] Bruix J (2021) Endpoints in clinical trials for liver cancer and their value in evidence-based clinical decision making: an unresolved Gordian knot. J Hepatol 74:1483–1488. 10.1016/j.jhep.2021.01.033 33556420 10.1016/j.jhep.2021.01.033

[11] Coudray N, Tsirigos A (2020) Deep learning links histology, molecular signatures and prognosis in cancer. Nat Cancer 1:755–757. 10.1038/s43018-020-0099-2 35122048 10.1038/s43018-020-0099-2 PMC11330634

[12] Jiang C, Chen K, Teng Y et al (2022) Deep learning–based tumour segmentation and total metabolic tumour volume prediction in the prognosis of diffuse large B-cell lymphoma patients in 3D FDG-PET images. Eur Radiol 32:4801–4812. 10.1007/s00330-022-08573-1 35166895 10.1007/s00330-022-08573-1

[13] Saillard C, Schmauch B, Laifa O et al (2020) Predicting survival after hepatocellular carcinoma resection using deep learning on histological slides. Hepatology 72:2000–2013. 10.1002/hep.31207 32108950 10.1002/hep.31207

[14] Shi J, Wang X, Ding G et al (2020) Exploring prognostic indicators in the pathological images of hepatocellular carcinoma based on deep learning. Gut 70:951–961. 10.1136/gutjnl-2020-320930 32998878 10.1136/gutjnl-2020-320930

[15] Liang J, Zhang W, Yang J et al (2023) Deep learning supported discovery of biomarkers for clinical prognosis of liver cancer. Nat Mach Intell 5:408–420. 10.1038/s42256-023-00635-3 10.1038/s42256-023-00635-3

[16] Chaudhary K, Poirion O, Lu L (2018) Deep learning-based multi-omics integration robustly predicts survival in liver cancer. Clin Cancer Res 24:1248–1259. 10.1158/1078-0432.ccr-17-0853 28982688 10.1158/1078-0432.ccr-17-0853 PMC6050171

[17] Isensee F, Jaeger PF, Kohl S et al (2020) nnU-Net: a self-configuring method for deep learning-based biomedical image segmentation. Nat Methods 18:203–211. 10.1038/s41592-020-01008-z 33288961 10.1038/s41592-020-01008-z

[18] Antonelli M, Reinke A, Bakas S et al (2022) The medical segmentation Decathlon. Nat Commun 13:4128. 10.1038/s41467-022-30695-9 35840566 10.1038/s41467-022-30695-9 PMC9287542

[19] Shao J, Zhong B (2003) Last observation carry-forward and last observation analysis. Stat Med 22:2429–2441. 10.1002/sim.1519 12872300 10.1002/sim.1519

[20] Tan M, Le QV (2019) EfficientNet: rethinking model scaling for convolutional neural networks. In: Proceedings of the 36th International Conference on Machine Learning, ICML, Long Beach, 9–15 June 2019.

[21] Hochreiter S, Schmidhuber J (1997) Long short-term memory. Neural Comput 9:1735–1780. 10.1162/neco.1997.9.8.1735 9377276 10.1162/neco.1997.9.8.1735

[22] Harrell FE, Lee KL, Califf RM, Pryor DB, Rosati RA (1984) Regression modelling strategies for improved prognostic prediction. Stat Med 3:143–152. 10.1002/sim.4780030207 6463451 10.1002/sim.4780030207

[23] Chambless LE, Diao G (2006) Estimation of time-dependent area under the ROC curve for long-term risk prediction. Stat Med 25:3474–3486. 10.1002/sim.2299 16220486 10.1002/sim.2299

[24] Selvaraju RR, Cogswell M, Das A, Vedantam R, Parikh D, Batra D (2017) Grad-CAM: visual explanations from deep networks via gradient-based localization. In: Proceedings of the IEEE international conference on computer vision, IEEE 618–626. 10.1109/iccv.2017.74

[25] He X, Li K, Wei R et al (2023) A multitask deep learning radiomics model for predicting the macrotrabecular-massive subtype and prognosis of hepatocellular carcinoma after hepatic arterial infusion chemotherapy. Radiol Med 128:1508–1520. 10.1007/s11547-023-01719-1 37801197 10.1007/s11547-023-01719-1 PMC10700409

[26] Meng Y, Zhang X, Zhang B et al (2023) Deep learning nomogram based on Gd-EOB-DTPA MRI for predicting early recurrence in hepatocellular carcinoma after hepatectomy. Eur Radiol 33:4949–4961. 10.1007/s00330-023-09419-0 36786905 10.1007/s00330-023-09419-0 PMC10289921

[27] Xu Y, Zhao J, Chen Q et al (2023) Contrastive learning for preoperative early recurrence prediction of hepatocellular carcinoma with liver CT image and tumor mask. In: 2023, 45th Annual international conference of the IEEE engineering in medicine & biology society (EMBC), IEEE, Piscataway. 10.1109/embc40787.2023.10340893 10.1109/EMBC40787.2023.10340893 38083328

[28] Zhang L, Xia W, Zhang Y et al (2020) Deep learning predicts overall survival of patients with unresectable hepatocellular carcinoma treated by transarterial chemoembolization plus sorafenib. Front Oncol. 10.3389/fonc.2020.593292 10.3389/fonc.2020.593292 PMC7556271 33102242

[29] Wei H, Zheng T, Zhang X et al (2024) MRI radiomics based on deep learning automated segmentation to predict early recurrence of hepatocellular carcinoma. Insights Imaging 15:120. 10.1186/s13244-024-01679-8 38763975 10.1186/s13244-024-01679-8 PMC11102894

[30] Pfister D, Núñez NG, Pinyol R et al (2021) NASH limits anti-tumour surveillance in immunotherapy-treated HCC. Nature 592:450–456. 10.1038/s41586-021-03362-0 33762733 10.1038/s41586-021-03362-0 PMC8046670

[31] Forner A, Llovet JM, Bruix J (2012) Hepatocellular carcinoma. Lancet 379:1245–1255. 10.1016/s0140-6736(11)61347-0 22353262 10.1016/s0140-6736(11)61347-0 PMC13537732

[32] Shimada M, Takenaka K, Gion T et al (1996) Prognosis of recurrent hepatocellular carcinoma: A 10-year surgical experience in Japan. Gastroenterology 111:720–726. 10.1053/gast.1996.v111.pm8780578 8780578 10.1053/gast.1996.v111.pm8780578

[33] Wada Y, Nakashima O, Kutami R, Yamamoto O, Kojiro M (1998) Clinicopathological study on hepatocellular carcinoma with lymphocytic infiltration. Hepatology 27:407–414. 10.1002/hep.510270214 9462638 10.1002/hep.510270214

[34] Wang H, Liu Y, Xu N et al (2022) Development and validation of a deep learning model for survival prognosis of transcatheter arterial chemoembolization in patients with intermediate-stage hepatocellular carcinoma. Eur J Radiol 156:110527. 10.1016/j.ejrad.2022.110527 36152524 10.1016/j.ejrad.2022.110527

[35] Kim H, Goo JM, Lee KH, Kim YT, Park CM (2020) Preoperative CT-based deep learning model for predicting disease-free survival in patients with lung adenocarcinomas. Radiology 296:216–224. 10.1148/radiol.2020192764 32396042 10.1148/radiol.2020192764

[36] Parmar C, Velazquez ER, Leijenaar RTH et al (2014) Robust radiomics feature quantification using semiautomatic volumetric segmentation. PLoS One 9:e102107. 10.1371/journal.pone.0102107 25025374 10.1371/journal.pone.0102107 PMC4098900

[37] Xu Y, Hosny A, Zeleznik R et al (2019) Deep learning predicts lung cancer treatment response from serial medical imaging. Clin Cancer Res 25:3266–3275. 10.1158/1078-0432.ccr-18-2495 31010833 10.1158/1078-0432.ccr-18-2495 PMC6548658

[38] De Frutos JP, Pedersen A, Pelanis E et al (2023) Learning deep abdominal CT registration through adaptive loss weighting and synthetic data generation. PLoS One 18:e0282110. 10.1371/journal.pone.0282110 36827289 10.1371/journal.pone.0282110 PMC9956065

[39] Hofmanninger J, Prayer F, Pan J, Röhrich S, Prosch H, Langs G (2020) Automatic lung segmentation in routine imaging is primarily a data diversity problem, not a methodology problem. Eur Radiol Exp 4:50. 10.1186/s41747-020-00173-2 32814998 10.1186/s41747-020-00173-2 PMC7438418

[40] Schütte K, Schinner R, Fabritius MP et al (2020) Impact of extrahepatic metastases on overall survival in patients with advanced liver dominant hepatocellular carcinoma: a subanalysis of the SORAMIC trial. Liver Cancer 9:771–786. 10.1159/000510798 33442545 10.1159/000510798 PMC7768116

