# Supplementary material for: CT-based multimodal deep learning for non-invasive overall survival prediction in advanced hepatocellular carcinoma patients treated with immunotherapy
Source: Insights Imaging. 2024 Aug 26;15:214. doi: 10.1186/s13244-024-01784-8 (PMC11347550; doi:10.1186/s13244-024-01784-8)
Supplement: Supplementary file 1 — ELECTRONIC SUPPLEMENTARY MATERIAL [file 13244_2024_1784_MOESM1_ESM.pdf]

CT-based Multimodal Deep Learning for Non-invasive Overall Survival Prediction in Advanced Hepatocellular Carcinoma Patients Treated with Immunotherapy

ELECTRONIC SUPPLEMENTARY MATERIAL

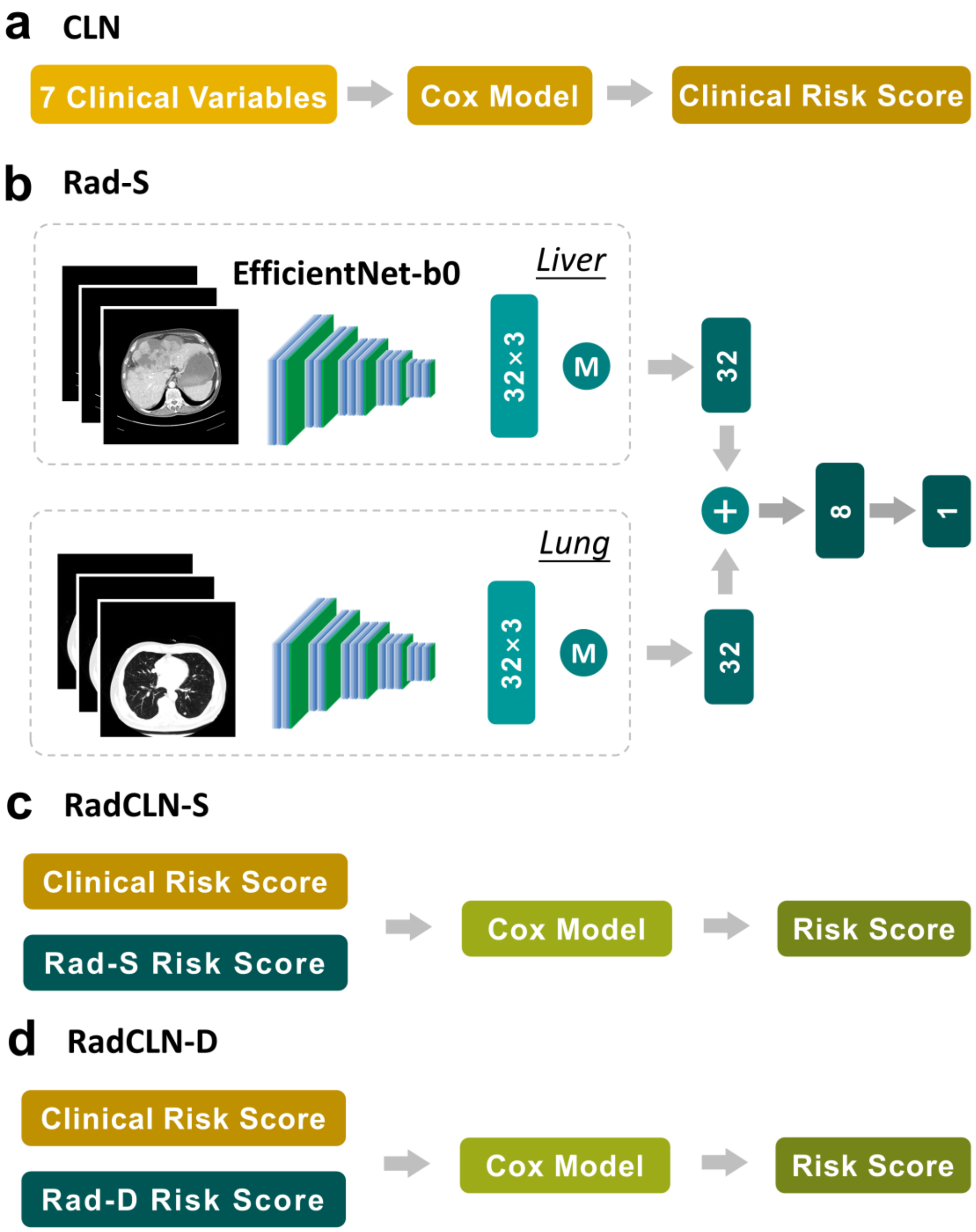

Figure S1: Model illustrations for CLN, Rad-S, RadCLN-S, and RadCLN-D.

Insights Imaging (2024) Xia Y, Zhou J, Xun X, et al.

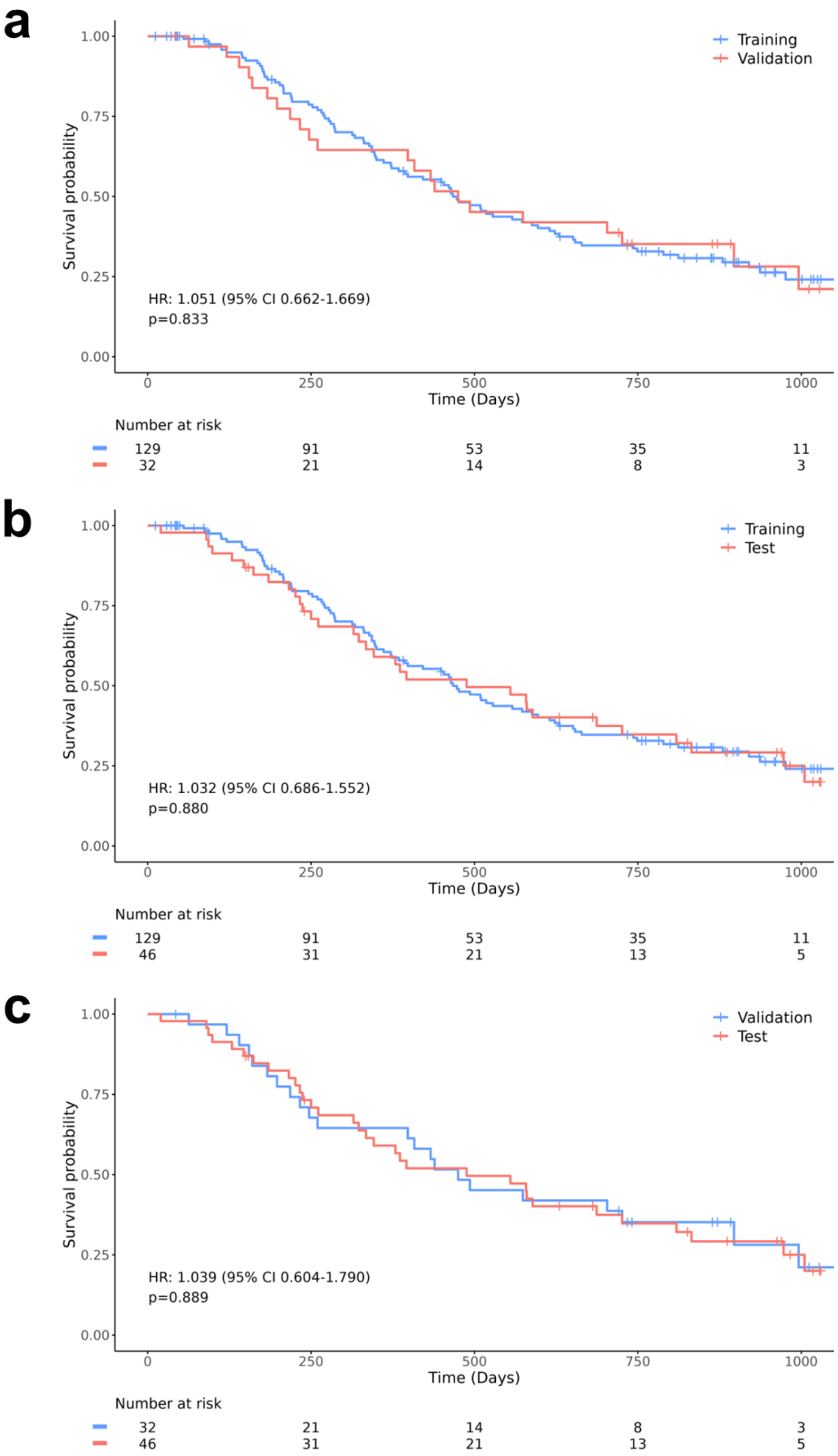

Figure S2: Comparisons of Kaplan-Meier curves on the training, validation, and test sets. There is no significant difference of the survival status among the three datasets.

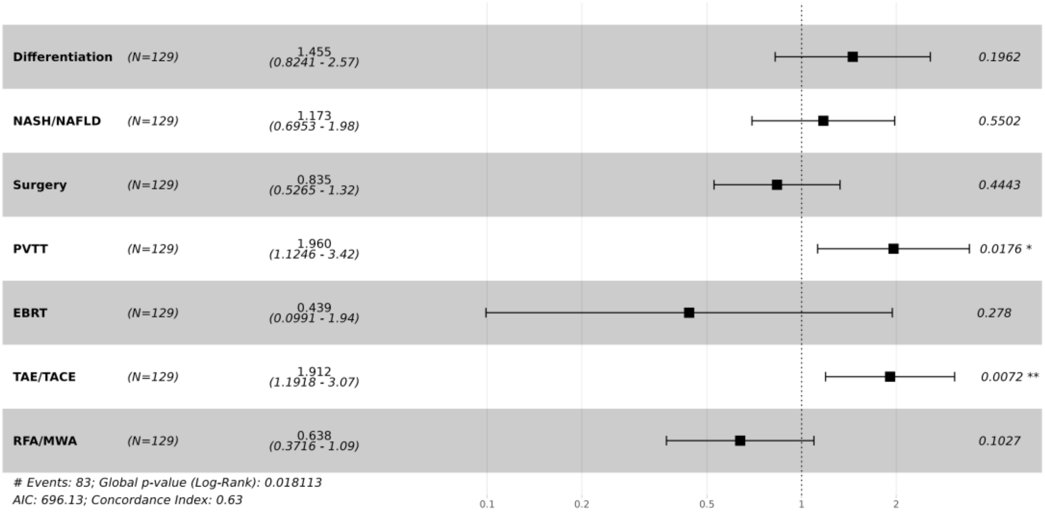

Figure S3: Forest plot for Cox model of the seven clinical variables. Differentiation, tumor histological differentiation type; NASH, non-alcoholic steatohepatitis; NAFLD, non-alcoholic fatty liver disease; PVTT, partial or complete portal vein tumor thrombosis; EBRT, external beam radiation therapy; TAE, transarterial embolization; TACE, transarterial chemoembolization, RFA, radiofrequency ablation; MWA, microwave ablation.

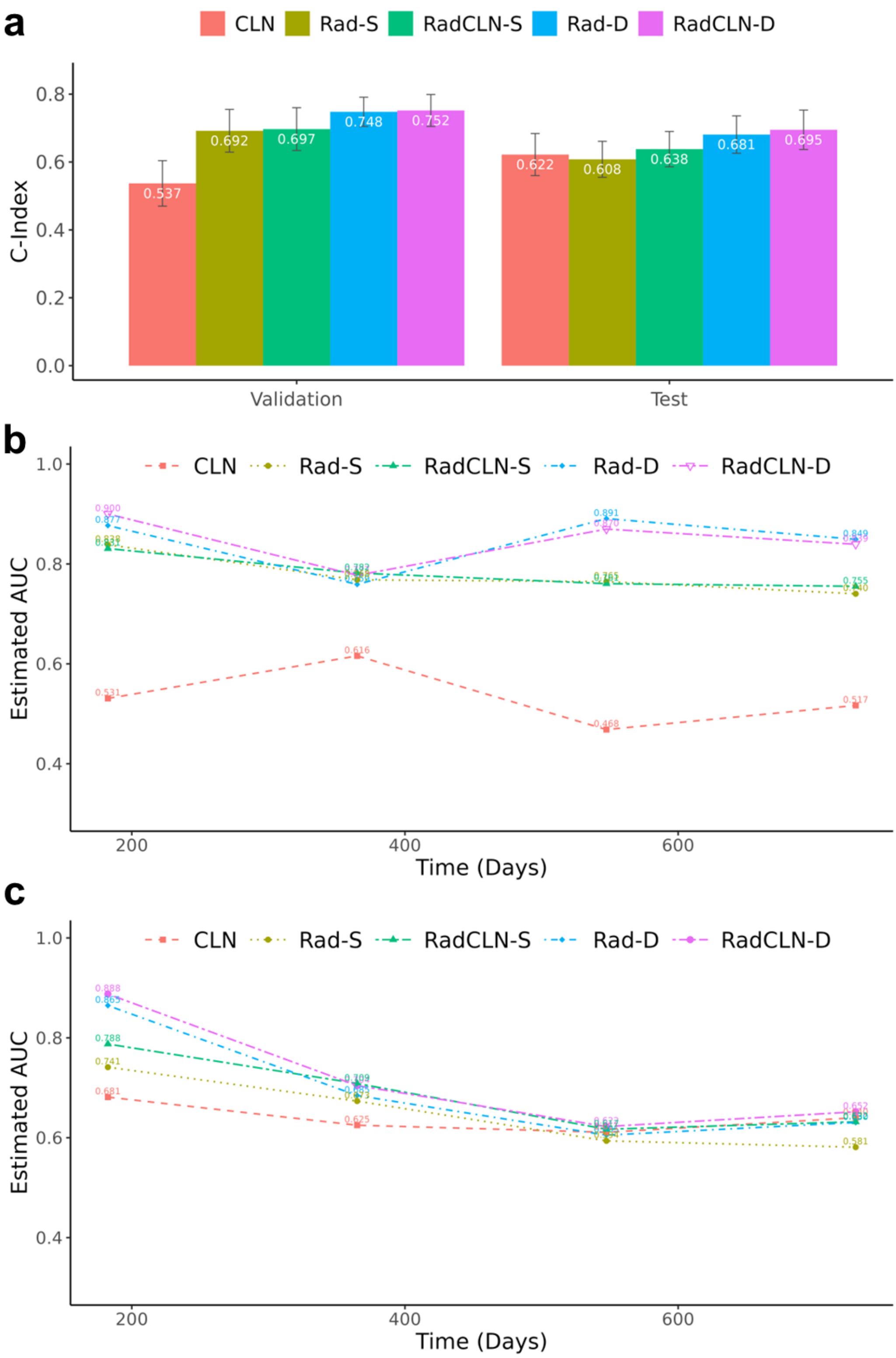

Figure S4: Overall survival prediction performances of the five models. Estimated c-index (a) (the error bar shows the 95% confidence interval), and estimated time-dependent AUCs of 0.5, 1, 1.5, and 2 years on the validation (b) and independent tests (c) were calculated.

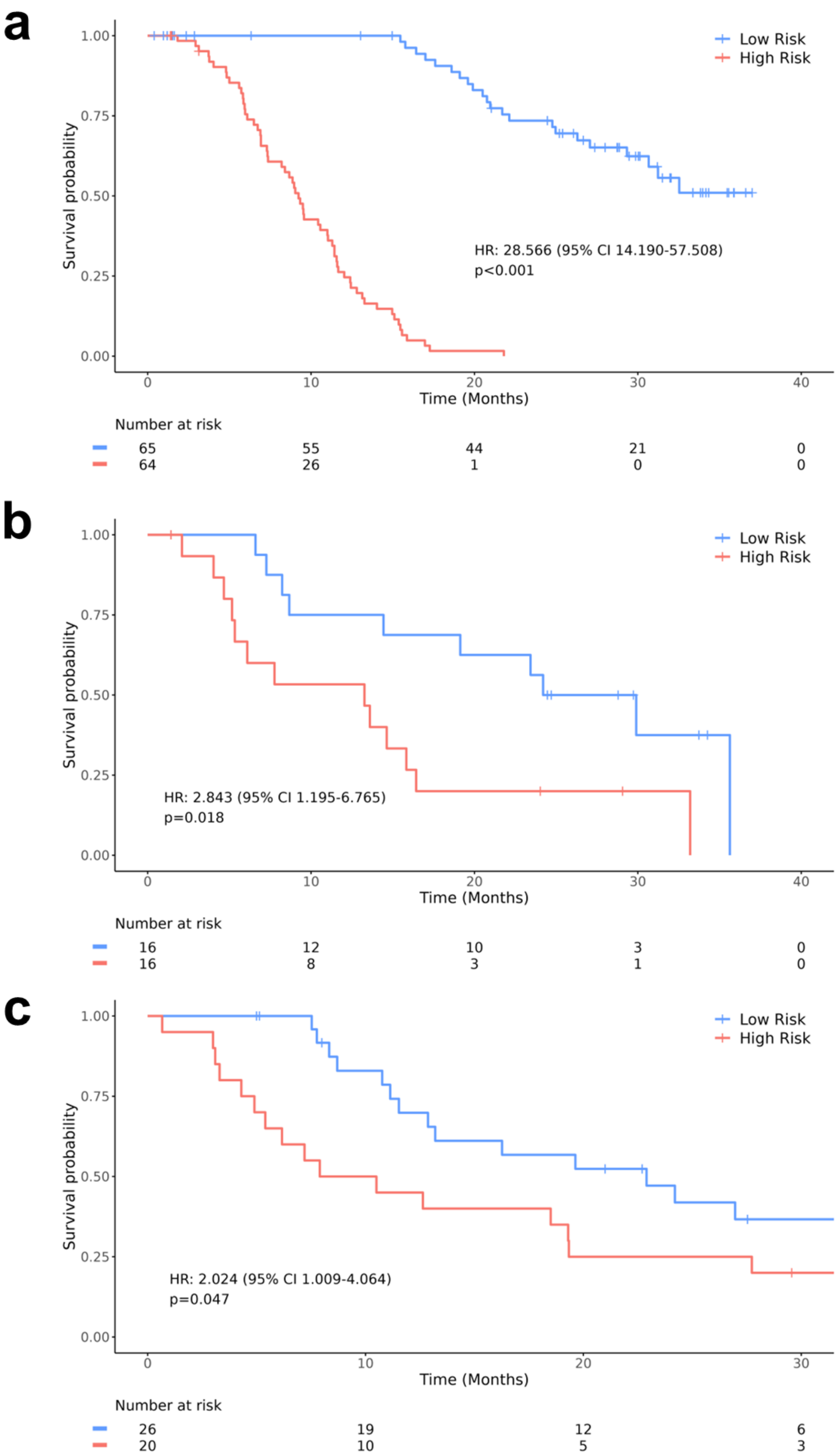

Figure S5: Kaplan-Meier survival estimates for the overall survival of Rad-D, stratified into low-risk and high-risk groups according to the median risk score in the training set.

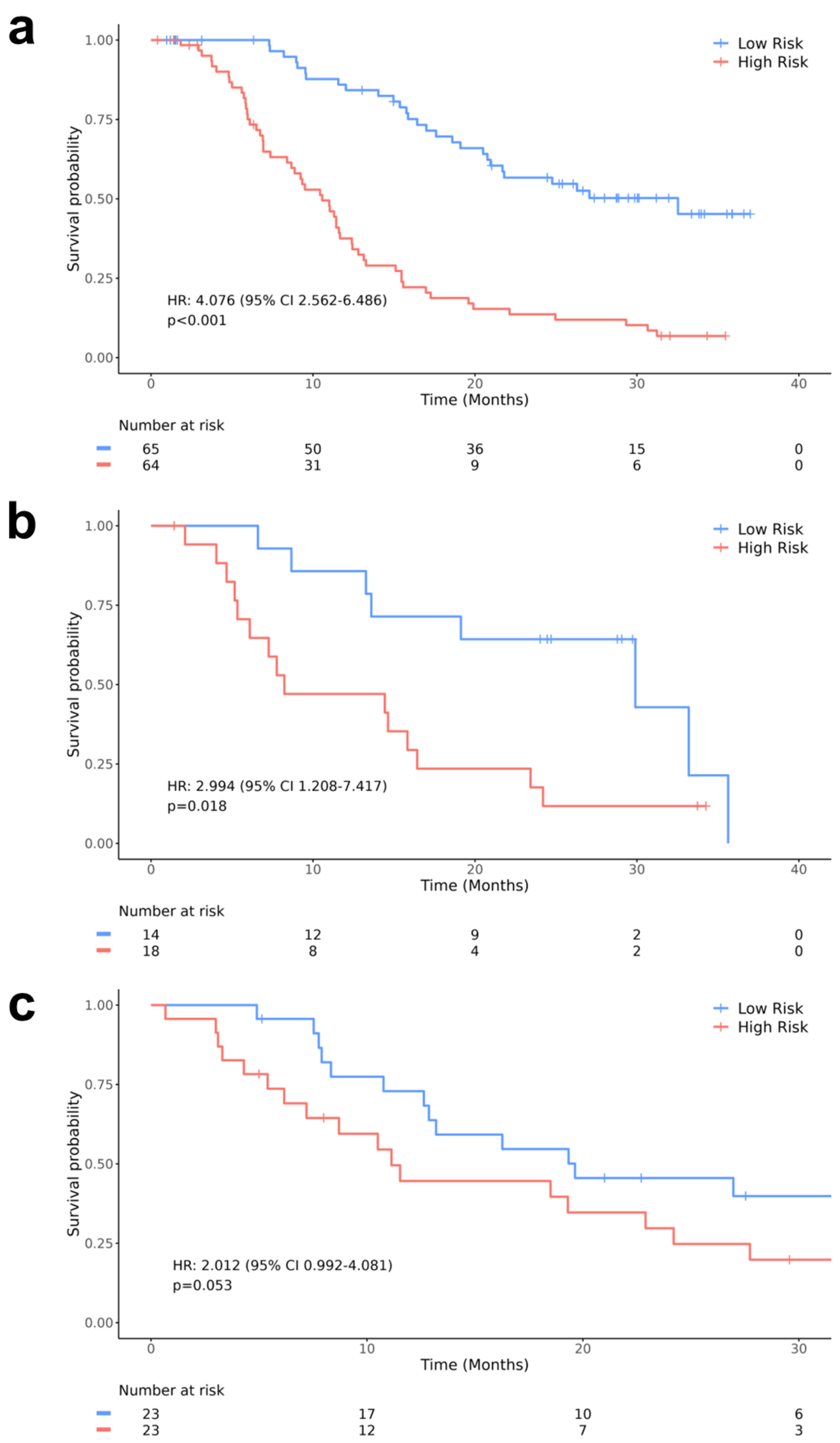

Figure S6: Kaplan-Meier survival estimates for the overall survival of RadCLN-S, stratified into low-risk and high-risk groups according to the median risk score in the training set.
